# Repositioning FDA Drugs as Potential Cruzain Inhibitors from *Trypanosoma cruzi*: Virtual Screening, In Vitro and In Vivo Studies

**DOI:** 10.3390/molecules22061015

**Published:** 2017-06-18

**Authors:** Isidro Palos, Edgar E. Lara-Ramirez, Julio Cesar Lopez-Cedillo, Carlos Garcia-Perez, Muhammad Kashif, Virgilio Bocanegra-Garcia, Benjamin Nogueda-Torres, Gildardo Rivera

**Affiliations:** 1 Unidad Académica Multidisciplinaria Reynosa-Rodhe, Universidad Autónoma de Tamaulipas, Carr. Reynosa-San Fernando, s/n, Reynosa 88779, México; isi_palos@hotmail.com; 2 Unidad de Investigación Biomédica de Zacatecas, Instituto Mexicano del Seguro Social (IMSS), Alameda Trinidad García de la Cadena, s/n, Zacatecas 98000, México; elarar0700@hotmail.com; 3 Departamento de Parasitología, Escuela Nacional de Ciencias Biológicas, Instituto Politécnico Nacional, Prolongación de Carpio y Plan de Ayala, s/n, Ciudad de México 11340, México; qbp.julio30@hotmail.com (J.C.L.-C); bnogueda@yahoo.com (B.N.-T.); 4 Laboratory of Pharmaceutical Biothecnology, Centro de Biotecnología Genómica, Instituto Politécnico Nacional, Boulevard del Maestro, s/n, Esq. Elías Piña, Reynosa 88710, México; carlosagp@hotmail.com (C.G.-P.); chkashif987@gmail.com (M.K.); vbocanegg@hotmail.com (V.B.-G.)

**Keywords:** drug repositioning, *Trypanosoma cruzi*, docking, cruzain, FDA drugs

## Abstract

Chagas disease (CD) is a neglected disease caused by the parasite *Trypanosoma cruzi*, which affects underdeveloped countries. The current drugs of choice are nifurtimox and benznidazole, but both have severe adverse effects and less effectivity in chronic infections; therefore, the need to discover new drugs is essential. A computer-guided drug repositioning method was applied to identify potential FDA drugs (approved and withdrawn) as cruzain (Cz) inhibitors and trypanocidal effects were confirmed by in vitro and in vivo studies. 3180 FDA drugs were virtually screened using a structure-based approach. From a first molecular docking analysis, a set of 33 compounds with the best binding energies were selected. Subsequent consensus affinity binding, ligand amino acid contact clustering analysis, and ranked position were used to choose four known pharmacological compounds to be tested in vitro. Mouse blood samples infected with trypomastigotes from INC-5 and NINOA strains were used to test the trypanocidal effect of four selected compounds. Among these drugs, one fibrate antilipemic (etofyllin clofibrate) and three β-lactam antibiotics (piperacillin, cefoperazone, and flucloxacillin) showed better trypanocidal effects (LC_50_ range 15.8–26.1 μg/mL) in comparison with benznidazole and nifurtimox (LC_50_ range 33.1–46.7 μg/mL). A short-term in vivo evaluation of these compounds showed a reduction of parasitemia in infected mice (range 90–60%) at 6 h, but this was low compared to benznidazole (50%). This work suggests that four known FDA drugs could be used to design and obtain new trypanocidal agents.

## 1. Introduction

Chagas disease (CD) is a neglected parasitic disease caused by the protozoan *Trypanosoma cruzi*. It represents a public health threat for under-developed countries in Latin America, where 350 million people are at risk for transmission and about 8 million are infected worldwide [1].

The infective pathway in human beings is with the bite of the haematophagous triatomine vector, which excretes trypomastigote infective forms near the bite site, allowing parasite penetration into the exposed tissue. During cell infection the trypomastigotes transform into reproductive amastigote forms, disrupting cells and allowing tissue dissemination of new trypomastigotes, causing a generalized invasion [2]. During acute phases of the infection, symptoms are commonly absent, but when the infection becomes chronic infected individuals develop cardiac, digestive or neurological symptoms, which are debilitating and potentially fatal [3].

During the pathogenic process, the parasite employs essential proteins to reach the intracellular environment of the host cell. Cruzain (Cz), a lysosomal cysteine protease, which is expressed in all life cycle stages of the parasite, has been implicated in the immune evasion, playing a relevant role in the host-parasite interaction [4]. One important fact, aside from its pathogenic role, is that this protein has no human homologue; as a result, Cz is an attractive drug target for developing new inhibitors. Actually, there are twenty five Cz structures deposited in the protein databank. Some of these crystal structures have been used for rational drug repositioning using chemoinformatics methodologies [5,6,7].

CD is currently treated with benznidazole and nifurtimox. These drugs cause severe adverse effects and are not useful for chronic infections [8]. Hence, there is a need for the development of new drugs for the treatment of CD. Several approaches can be considered for developing new drugs. One interesting alternative is drug repositioning. This method can be used as a short-path approach because their pharmacokinetics and pharmacodynamics are well known for specific drug-molecules. This makes them acceptable for regulatory health authorities, such as the Food and Drug Administration (FDA) [9]. Therefore, when a new therapeutic use is identified for a known drug molecule, it can swiftly progress to clinical trials [10], shortening the time of experimental validation, and with more probability for success on the pharmaceutical market than new drugs [11]. For example, the drug sildenafil, which was initially designed for the treatment of hypertension and ischemic heart disease, was later approved for the treatment of erectile dysfunction, representing a successful history of drug repositioning.

In the present work, we report a computational drug virtual screening protocol to identify FDA drugs as potential Cz inhibitors. Furthermore, we performed in vitro evaluations and short-term in vivo evaluations of the identified FDA drugs.

## 2. Results and Discussion

### 2.1. Computational Analysis

**Figure 1:**
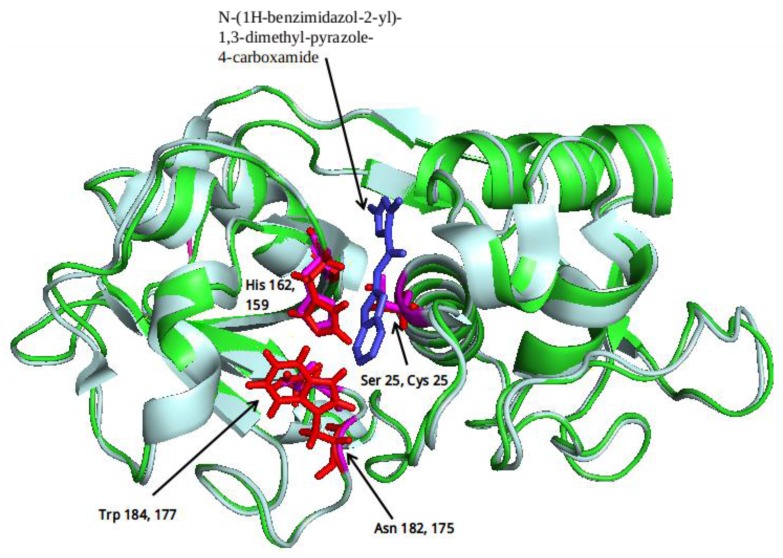
Structural comparison of the 4W5B protein with 1F2A protein. In green the 4W5B protein, in pale cyan the 1F2A protein that contain the known catalytic triad. The catalytic amino acids indicated by arrows are colored as follow: 1F2A in magenta and 4W5B in red. The compound *N*-(1H-benzimidazol-2-yl)-1,3-dimethyl-pyrazole-4-carboxamide is shown in blue.

Compound *N*-(1H-benzimidazol-2-yl)-1,3-dimethyl-pyrazole-4-carboxamide included in the 4W5B pdb file (Figure 1) was used as a control in the database.

The structure-based virtual screening predicted 1678 FDA approved drugs with a higher negative score than the control (−6.1 kcal/mol) (Supplementary Materials 1). From this filtration, 33 top compounds were selected that showed the lowest negative vina score in a range of −8.4 to −8.9 kcal/mol. These compounds were further re-ranked, taking into account the consensus score (mean of Z-scores) calculated from the Z-scores for the vina, X-score and drugscore scoring functions (Table 1). This step was applied because the consensus score increases the probability of selecting active compounds for further experimental validations [12].

A substantial heterogeneity of chemical structures was observed for the best-ranked compounds, because among these potential Cz inhibitors exist antibiotics, antidiabetics, antipsychotics, antilipemics, antibiotics, anti-neoplastics, and drugs without known commercial information and pharmacological use (Table 1). Thus, to understand the relationship of these chemical compounds (regarding their ranked position) and to help us choose the compounds for subsequent in vitro analysis, a clustering method was applied.

**Figure 2:**
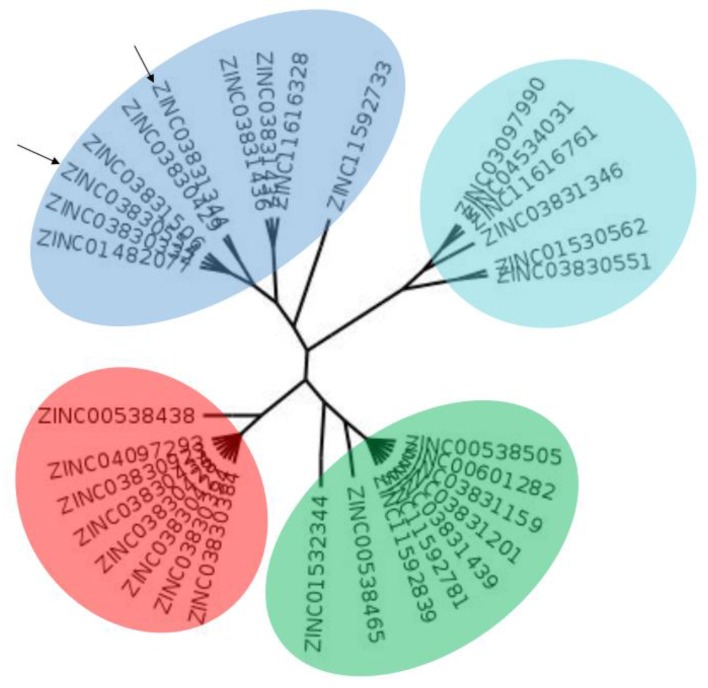
Clustering pattern based on ligand contact amino acid showing the four major groups. In cyan, group 1, in blue, group 2, in red, group 3, and in green, group 4. Arrows indicate the compound ZINC03830554 and ZINC03831344 mentioned in the text.

The clustering method takes into account the interaction of the compounds with the amino acids of the active site, and the analysis was focused on the inspection of the ligand contact with the essential amino acids of the catalytic triad Cys25, His159, and Asn175, and the well conserved Trp 177 (Ser25, His162, Asn182, and Trp184 in 4W5B pdb file, Figure 1) for the cysteine protease families [13]. The matrix of ligand amino acid contact (Supplementary Materials 2) generated by the AuPosSOM software showed that the 33 compounds interact with most of the catalytic residues mentioned above (except Asn182), indicating that each compound contains key chemical elements for the inhibition of Cz. Moreover, the clustering pattern of the tree showed four groups (Figure 2) and within these, drugs with good scoring and low scoring; for example, group 2 contains the best-ranked compound ZINC03830554 (Z-mean= −1.391) without available information, and the compound ZINC03831344 (Z-mean= −0.352), which is the antibiotic piperacillin with known biological activity but with a low consensus score (Figure 1), is located in the same group. Based on this observation, it was decided to test those compounds that were related with the best-ranked compound, or which were well ranked and have pharmacological and commercial information. Therefore, four compounds were selected: the antilipemic etofyllin clofibrate (ZINC00538438, Z-mean= −0.609) from group 3, the antibiotics flucloxacillin sodium (ZINC01532344, Z-mean= 0.287) from group 4, piperacillin sodium (ZINC03831344, Z-mean= −0.352), and cefoperazone sodium (ZINC03830429, Z-mean= −0.137) from group 2. Group 1 was avoided because it is composed of antibiotic analogues of the previous chosen compounds, such as the piperacillin analogue (ZINC03831346, Z-mean= −0.786).

The known FDA information for the selected compounds is as follows: Etofyllin clofibrate [14], belonging to the fibrate class of drugs, is used for diminishing triglyceride levels in blood through activation of the transcription factor PPAR-alfa that oxidizes fatty acids and stimulates lipoprotein lipase [15]. The three antibiotics are known β-lactam derivatives of the class cephalosporin, which act through inhibition of transpeptidase, essential for bacteria cell wall synthesis [16].

**Figure 3:**
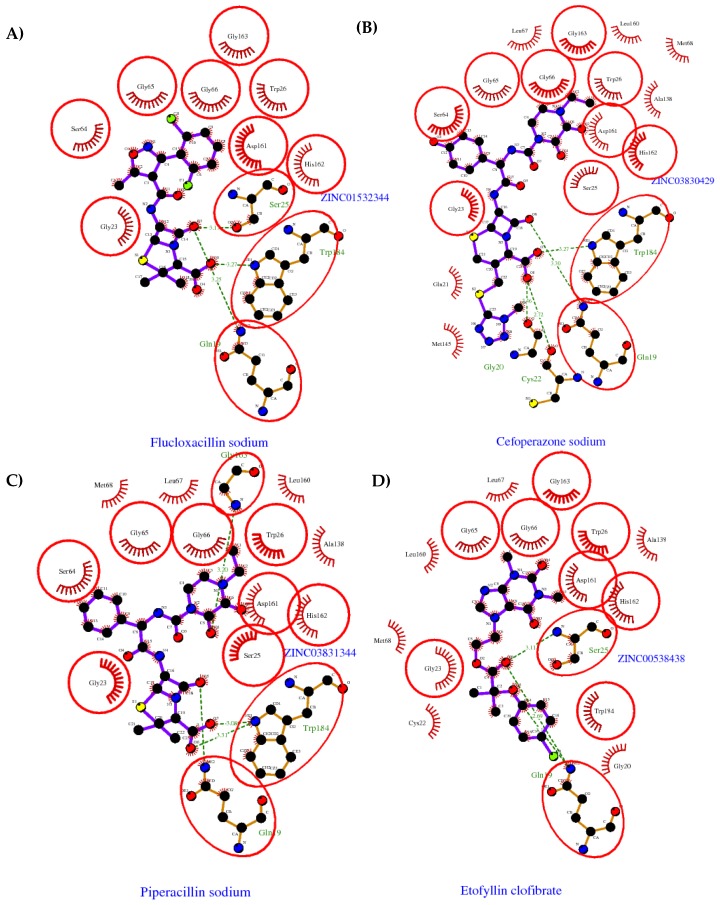
The four selected compounds docked in the 4W5B Cz active sites. (**A**) Flucloxacillin sodium, (**B**) Cefoperazone sodium, (**C**) Piperacillin sodium, (**D**) Etofyllin clofibrate. In the four figures arcs with red lines represent amino acid hydrophobic contacts, green dashed lines represent hydrogen bonds, the red circles represent the shared amino acids. The image was produced with LigPlot software [17].

The four selected compounds interact with the catalytic amino acids Ser25, His162 and Trp184 (Figure 3A–D). Among them, only the flucloxacillin (Figure 3A) and etofyllin (Figure 3D) participate in nucleophilic covalent reaction with the catalytic Ser25 amino acid.

Three studies exist that use a computational protocol for repositioning FDA drugs as Cz inhibitors. The first study predicted the antidiabetic bromocriptine and the antiarrhythmic amiodarone as potential inhibitors, and further enzymatic studies confirmed the inhibitory effects on Cz. In vitro studies showed trypanocidal effects on the epimastigotes of the *T. cruzi* strain [5]. The second study predicted levothyroxine, a drug used for hypothyroidism, as a Cz inhibitor. This was also confirmed by enzymatic studies. The in vitro experiments also showed antiproliferative effects on epimastigotes [6]. The third report predicted the antileprosy drug, clofazimine, and the calcium channel blocker benidepine, as potential inhibitors. The two compounds were further tested in vitro, showing inhibitory effects on epimastigotes and trypomastigotes and on Cz protein during enzymatic studies [7]. In line with the previous reports, in the present study we also employed a computational methodology to predict potential Cz inhibitors and their trypanocidal effects are presented in the next section.

### 2.2. Anti-Trypanosoma cruzi Activity

The four compounds were evaluated for their direct trypanocidal effects on blood samples infected with trypomastigotes from the NINOA (MHOM/MX/1994/NINOA, was obtained from a patient with acute CD) and INC-5 (MHOM/MX/1994/INC-5, was obtained from a patient in the chronic phase of the disease) strains (Table 2). The drugs tested showed better trypanocidal effects than the drugs of reference, benznidazole and nifurtimox. The compounds flucloxacillin, piperacillin, and cefoperazone were mostly active on the INC-5 strain, and among them, piperacillin was the most active (LC_50_= 15.8 ± 1.4 μg/mL). The compounds etofyllin clofibrate, flucloxacillin, and cefoperazone were mostly active on the NINOA strain, and among them, etofyllin clofibrate was the most active (LC_50_= 18.4 ± 0.9 μg/mL). LC_50_ was not measured for etofyllin clofibrate on INC-5 strain and piperacillin sodium on NINOA strain because they showed trypanocidal activity <50% at 50 μg/mL compared with the reference drugs.

**Figure 4:**
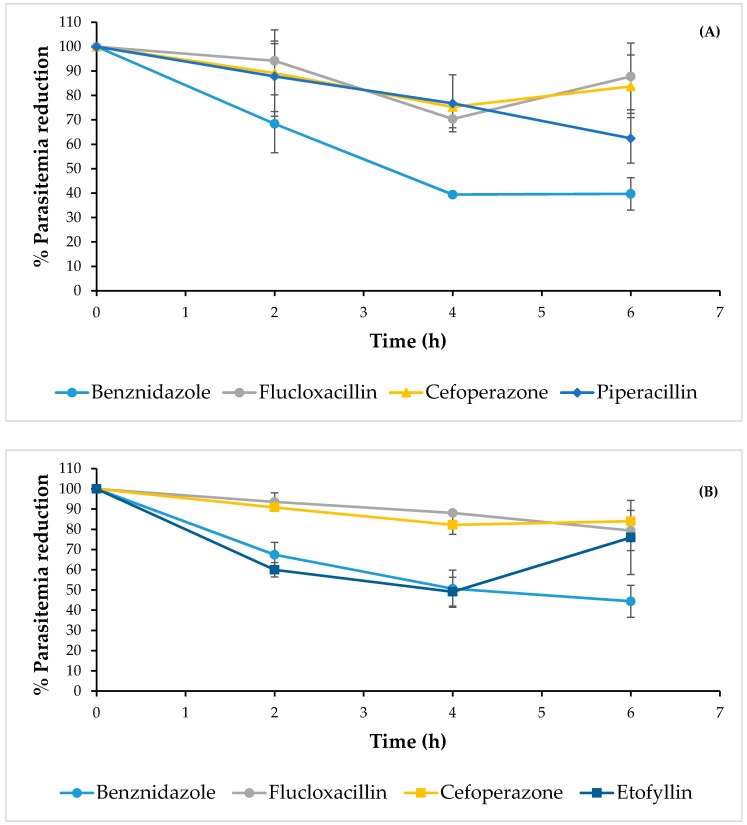
Effects of the compounds in reducing parasites on mice infected with INC-5 (**A**) and NINOA (**B**) strains from *T. cruzi* over a period of 6 h.

Since these compounds are already used in humans, a short-term in vivo experiment was carried out in a mice infected model to extrapolate possible trypanocidal effects in humans. During the in vivo evaluations, three compounds (flucloxacillin, cefoperazone and etofyllin) maintained trypanocidal effects on mice infected with NINOA strains. In this mice group, it was interesting that the antilipemic etofyllin clofibrate showed a 60% reduction of parasites at 4 h, a result comparable with benznidazole (Figure 4B); however, parasites rose again at 6 h. In the case of the mice group infected with INC-5 strains, three compounds (flucloxacillin, cefoperezone and piperacillin) were less effective in comparison with benznidazole (reduction of 50% at 6 h); although piperacillin showed a decrease of parasitemia close to 70% at 6 h (Figure 4A). These results suggest that piperacillin and etofyllin clofibrate could be potential Cz inhibitors, but further confirmatory enzyme inhibition analyses are necessary. Aside from the latter limitation, the results obtained reinforce the advancement in drug-repositioning research for the treatment new diseases, as in our case, CD.

## 3. Materials and Methods

### 3.1. Database Creation and Docking Protocol

The structure-based virtual screening was carried out as previously described [17]. First, 3180 FDA drugs (approved and withdrawn) were retrieved from the ZINC website [18] (Supplementary Materials 3). Those 3180 compounds were used to create a database of compounds using the prepare_ligand4.py python script from AutoDockTools [19]. This script allows the merging of non-polar hydrogens, adding Gasteiger charges, and setting up rotable bonds for each ligand in order to produce the pdbqt file format necessary for the AutoDock Vina software, which was used for the docking process [20]. The Cz (PDB ID 4W5B) protein file [21] was retrieved from the Protein Data Bank. This protein has the catalytic triad Cys25, His159, Asn175, and the well-conserved Trp177 for cysteine proteases families in the amino acid positions Ser25, His162, Asn182 and Trp184 (Figure 1). We decided to use this protein because the binding pose of the ligand *N*-(1H-benzimidazol-2-yl)-1,3-dimethyl-pyrazole-4-carboxamide was the most similarly reproducible (rmsd= 1.096) with Autodock Vina during the search for the optimal size grid-box required for the docking process; thus 4W5B was prepared as a receptor by removing the ligands and water molecules. Then, polar hydrogens, Gasteiger charges, and the Vina configuration file were assigned using the AutoDock Tools interface.

The first docking processes were carried out with the compound *N*-(1H-benzimidazol-2-yl)-1,3-dimethyl-pyrazole-4-carboxamide (thereafter, the control) in order to determine the size of search spaces on the active site for Cz. First, the control was placed on the active site and several rounds of dockings were carried out to increase the size of search space. Finally, the size of search spaces in each dimension was 14 Å, and its center was 36.722, 41.543, and 8.499 for x, y and z, respectively. The binding energy obtained for the control was introduced in the ligand dataset, and was setup as the cutoff value to select potential inhibitors. Compounds with high binding-energy values (according to the vina scoring function) above the cutoff value were considered inactive compounds (inactive compounds set); therefore, the lowest top-binding energy compounds were considered as potential inhibitors. From this preliminary set of inhibitors the top ranked potential inhibitors were chosen to perform a consensus score (Z-mean) using three scoring-functions: vina, DrugScore [22] and X-score [23], which were arranged by group. To determine the latter two scores, the docking coordinates produced by vina were used to feed the DrugScore and X-score programs, because neither scoring-function is included in a specific docking program. For each one of these three scoring groups the Z-scores were calculated, taking into account the scoring value obtained for each compound within the group, minus the mean value for the entire scoring group, and divided by the standard deviation as previously reported [24]. The Z-scores obtained for each compound within the groups were used to calculate the final consensus score, i.e., the Z-mean (the average of Z-scores).

### 3.2. Clustering and Ligand-Amino Acid Contact Analysis

Ligand-contact analysis was carried out for the top-ranked compounds that resulted from the docking study. Clustering of ligands with essential amino acids of the receptor was performed with the software AuPosSOM (Automatics analysis of poses using SOM) [25]. The clustering process involved three steps: (1) first, a Kohonen self-organizating map (SOM) was trained using ligand-amino acid descriptors; (2) then an unsupervised cluster analysis was performed; and (3) finally, a Newick tree file was generated for visual analysis. The tree was prepared using Fig tree software (http://tree.bio.ed.ac.uk/software/figtree/).

### 3.3. In Vitro Evaluation

Three drugs were purchased from Sigma Aldrich (Toluca, Mexico, code: 32353, C4292, P8396), and one from Enamine (Kiev, Ukraine; EN300), to perform the in vitro evaluation, as follows. We used *T. cruzi* bloodstream trypomastigotes from INC-5 and NINOA strains obtained by cardiac puncture from infected NIH mice at the peak of infection and adjusted to 1 × 10^6^ blood forms/mL. The purchased compounds were dissolved in dimethyl sulfoxide (DMSO) and mixed with infected blood to a final concentration of 5 μg/mL. The final concentration of DMSO in the culture medium remained below 1%. A solution of DMSO/H_2_O (1:99) was used as a negative control. The test was performed three times on 96-well microplates (Biofil JET) containing 195 μL of infected blood and 5 μL of the compound per well. The plates were incubated for 24 h at 4 °C to avoid a change of phase to epimastigote [26]. Bloodstream trypomastigotes were quantified by the Brener method [27]. Briefly, 5 μL of blood was placed on slides, covered with a coverslip, and the flagellates were examined with an optical microscope at 40× magnification. Anti-*T. cruzi* activity was expressed as lysis percentage by comparing the remaining trypomastigotes in each concentration with respect to the negative control group. Each assay was performed three times for each *T. cruzi* strain. LC_50_ values for dose-response were determined using Probit analysis and the results were expressed as mean ± standard deviation (SD).

### 3.4. In Vivo Study of the Effects of the Compounds on T. cruzi

Short-term in vivo evaluations were performed following the Filardi and Brener as well as the Romanha methodology [28,29]. Briefly, groups of five NIH female mice (20–25 g) were inoculated intraperitoneally with 2 × 10^5^ bloodstream trypomastigotes of *T. cruzi* INC5 and NINOA strains. The four compounds, including reference drug benznidazole, were suspended in 4% arabic gum (Sigma Aldrich, Toluca, Mexico). At the peak of parasitemia (19th and 24th days), mice were orally administered a single dose of 100 mg/kg of each compound. The controls were treated only with the vehicle. Parasitemia was measured before, and 2, 4 and 6 h after compound administration, using blood from the tail. The percentage of reduction of parasitemia was calculated microscopically by comparing the number of blood trypomastigotes obtained at each interval of time after compound administration with that found before treatment. Animal experiments were performed according to our country law Norma Oficial Mexicana (NOM-062-Z00-1999) published on 22 August 2009.

## 4. Conclusions

In this report, we used a structure-based virtual screening method for 3180 FDA-approved and/or withdrawn drugs against Cz protein from *T. cruzi*. The computational method includes the combination of a consensus scoring and clustering method to help us choose the best compounds for in vitro testing and subsequent in vivo evaluations. The in vitro evaluation on trypomastigotes from INC-5 and NINOA strains suggested that four FDA drugs could be used as potential Cz inhibitors, because the three antibiotics and the antilipemic showed better trypanocidal effects than the reference drugs on both strains. The short-term in vivo evaluation evidenced that two compounds (etofillyn clofibrate and piperacillin) conserved the inhibitory effects, albeit low effects, in comparison with benznidazole. To the best of our knowledge, this is the first study that argues the possible use of an antibiotic and an antilipemic for development of new antitrypanocidal agents.

## Figures and Tables

**Table 1:** The Z-mean values used for ranking the potential Cz inhibitors and their FDA indication.

ZINC ID	Z-Mean	Compound Structure	FDA Indication *
ZINC03830554	−1.391	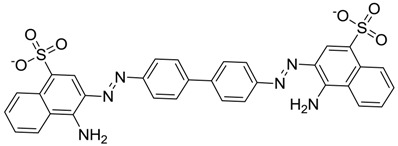	NAI
ZINC03831439	−0.864	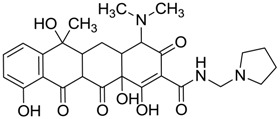	Antibiotic (rolitetracycline)
ZINC03831201	−0.846	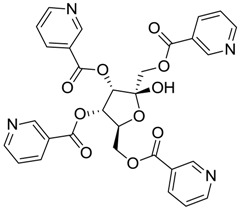	NAI (analogue fructofuranose tetranicotinate)
ZINC03831346	−0.786	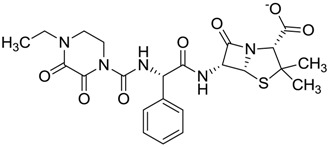	Antibiotic (piperacillin sodium)
ZINC03831506	−0.768	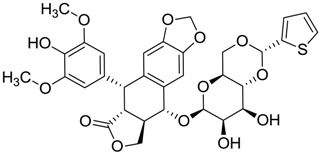	Antineoplastic (analogue teniposide)
ZINC00538438	−0.609	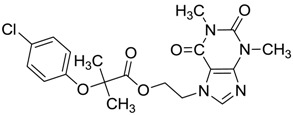	Antilipemic (etofylline clofibrate)
ZINC03830384	−0.596	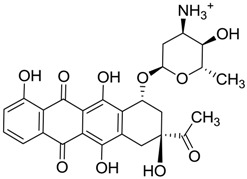	Antineoplastic (carubicin)
ZINC03830923	−0.504	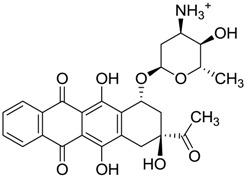	Antineoplastic (idarubicin)
ZINC11592781	−0.472	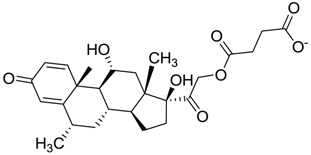	Anabolic steroid (analogue methylpredinisoloe)
ZINC00538505	−0.402	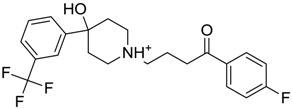	Antypsychotic (trifluperidol)
ZINC03831344	−0.352	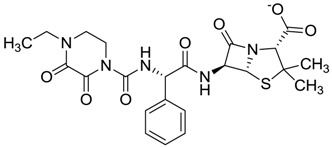	Antibiotic (piperacillin sodium)
ZINC11592839	−0.346	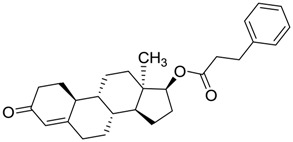	Anabolic steroid (analogue nandrolone phenilpropionate)
ZINC11592733	−0.274	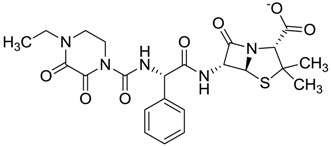	Antibiotic (analogue ampicillin)
ZINC03830427	−0.171	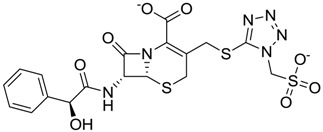	Antibiotic (analogue cefonicid)
ZINC03830429	−0.137	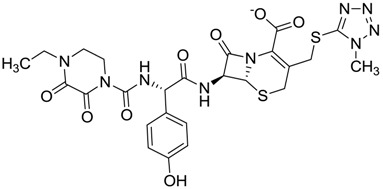	Antibiotic (cefoperazone)
ZINC00601282	−0.071	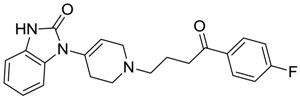	NAI
ZINC01482077	−0.039	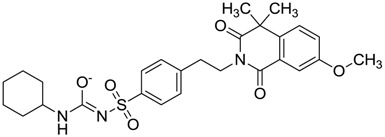	Antidiabetic (gliquidone)
ZINC03830428	−0.033	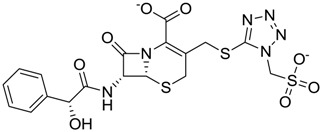	Antibiotic (cefonicid sodium)
ZINC03830332	−0.013	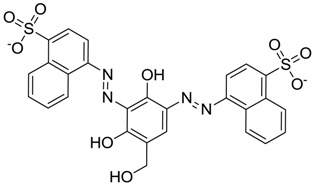	Dye (analogue chocolate brown)
ZINC03830434	0.017	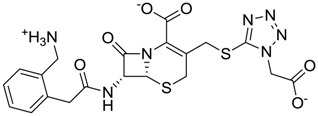	Antibiotic (ceforanide)
ZINC03831159	0.154	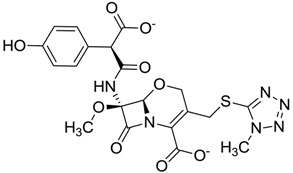	Antibiotic (analogue moxalactam disodium) *
ZINC00538465	0.235	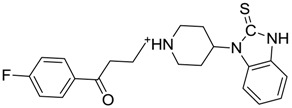	NAI
ZINC11616761	0.276	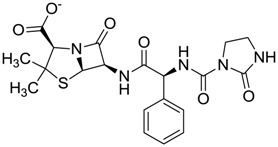	Antibiotic (analogue ampicillin)
ZINC03831436	0.278	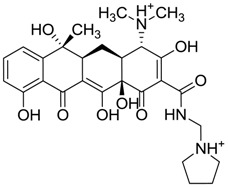	Antibiotic (rolitetracycline)
ZINC01532344	0.287	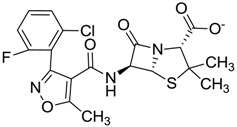	Antibiotic (flucloxacillin sodium)
ZINC03831437	0.328	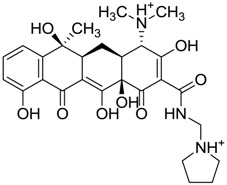	Antibiotic (rolitetracycline)
ZINC11616328	0.436	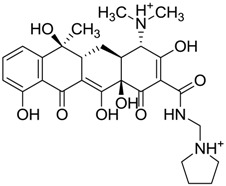	Antibiotic (rolitetracycline)
ZINC03830394	0.441	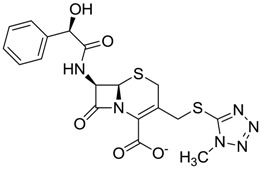	Antibiotic (cefamandole)
ZINC01530562	0.455	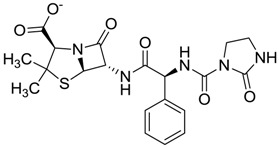	Antibiotic (analogue mezlocilline)
ZINC04534031	0.466	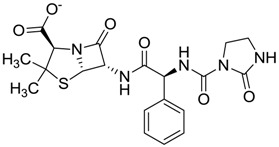	Antibiotic (analogue mezlocilline)
ZINC03097990	0.544	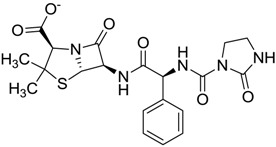	Antibiotic (analogue mezlocilline)
ZINC04097293	0.610	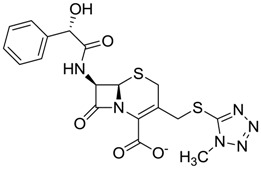	Antibiotic (cefamandole)
ZINC03830551	1.080	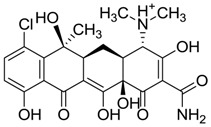	Antibiotic (analogue tetracycline)

* FDA indication based on ZINC database and search query on Chemspider, Google, PubChem, and ChEMBL. NAI = Not Available Information.

**Table 2:** LC_50_ of the FDA drugs on *T. cruzi* strains.

Name	Clinical Use	% Lysis on INC-5 at 50 µg/mL	LC_50_ (µg/mL) on INC-5	% Lysis on NINOA at 50 µg/mL	LC_50_ (µg/mL) on NINOA
Etofyllin clofibrate	Antilipemic	21	ND	60	18.4 ± 0.9
Flucloxacillin sodium	Antibiotic	61	26.1 ± 1.4	81	23.2 ± 1.4
Piperacillin sodium	Antibiotic	65	15.8 ± 1.4	43	ND
Cefoperazone sodium	Antibiotic	71	23 ± 1.8	64	25.8 ± 0.7
Benznidazole	Antichagasic	56	40.6 ± 2.4	69	46.6 ± 1.9
Nifurtimox	Antichagasic	51	46.7 ± 5.2	63	33.1 ± 1.3

ND: Not determined, SD in % lysis was all times <5.0%.

## Supplementary Materials

The following are available online at www.mdpi.com/1420-3049/22/6/1015, Supplementary Materials 1: the best ranked compounds according to the vina scoring function, Supplementary Materials 2: the matrix of ligand amino acid contact produced with the AuPosSOM software. Supplementary Materials S3: the 3180 FDA-approved and withdrawn drugs obtained from ZINC database.
